# Genome-wide RNAi screen for genes regulating glycolytic response to vemurafenib in BRAF^V600^ melanoma cells

**DOI:** 10.1038/s41597-020-00683-z

**Published:** 2020-10-12

**Authors:** Lorey K. Smith, Tiffany Parmenter, Cathryn M. Gould, Piyush B. Madhamshettiwar, Karen E. Sheppard, Kaylene J. Simpson, Grant A. McArthur

**Affiliations:** 1 Cancer Research Division, Peter MacCallum Cancer Centre, Parkville, Australia; 2 Victorian Centre for Functional Genomics, Peter MacCallum Cancer Centre, Parkville, Australia; 3 Sir Peter MacCallum Department of Oncology, University of Melbourne, Parkville, VIC 3010 Australia

**Keywords:** Cancer metabolism, Targeted therapies, Melanoma

## Abstract

Identification of mechanisms underlying sensitivity and response to targeted therapies, such as the BRAF inhibitor vemurafenib, is critical in order to improve efficacy of these therapies in the clinic and delay onset of resistance. Glycolysis has emerged as a key feature of the BRAF inhibitor response in melanoma cells, and importantly, the metabolic response to vemurafenib in melanoma patients can predict patient outcome. Here, we present a multiparameter genome-wide siRNA screening dataset of genes that when depleted improve the viability and glycolytic response to vemurafenib in BRAF^V600^ mutated melanoma cells. These datasets are suitable for analysis of genes involved in cell viability and glycolysis in steady state conditions and following treatment with vemurafenib, as well as computational approaches to identify gene regulatory networks that mediate response to BRAF inhibition in melanoma.

## Abstract (summary)

**Table:** 

Measurement(s)	cell viability • glycolytic process
Technology Type(s)	immunofluorescence microscopy assay • absorbance
Factor Type(s)	drug treatment • gene silencing
Sample Characteristic - Organism	Homo sapiens

Machine-accessible metadata file describing the reported data: 10.6084/m9.figshare.12885593

## Background & Summary

Oncogene targeted therapies have significantly improved clinical outcomes for cancer patients, however response to these therapies is limited by development of resistance. In the setting of BRAF mutant melanoma, inhibition of BRAF signalling results in oncogenic network rewiring that allows cellular adaptation and drug tolerance, and this often precedes development of resistance. In both preclinical and clinical studies of melanoma, drug induced adaptation reprograms metabolism, and importantly, sensitivity to inhibitors of mutated BRAF^V600^ correlates with glycolytic response in pre-clinical^1^ and clinical studies^2^. Notably, alterations in cellular metabolism is a hallmark of cancer, although how targeted therapy reprograms metabolism and the role this plays during the adaptive response and development of resistance has received much less attention. The study described herein aimed to elucidate mechanisms underlying response to BRAF inhibition using a functional genomics screen to identify genes whose depletion enhanced the viability and glycolytic response of BRAF^V600^ melanoma cells to the BRAF inhibitor (BRAFi) vemurafenib (vem).

**Fig. 1:**
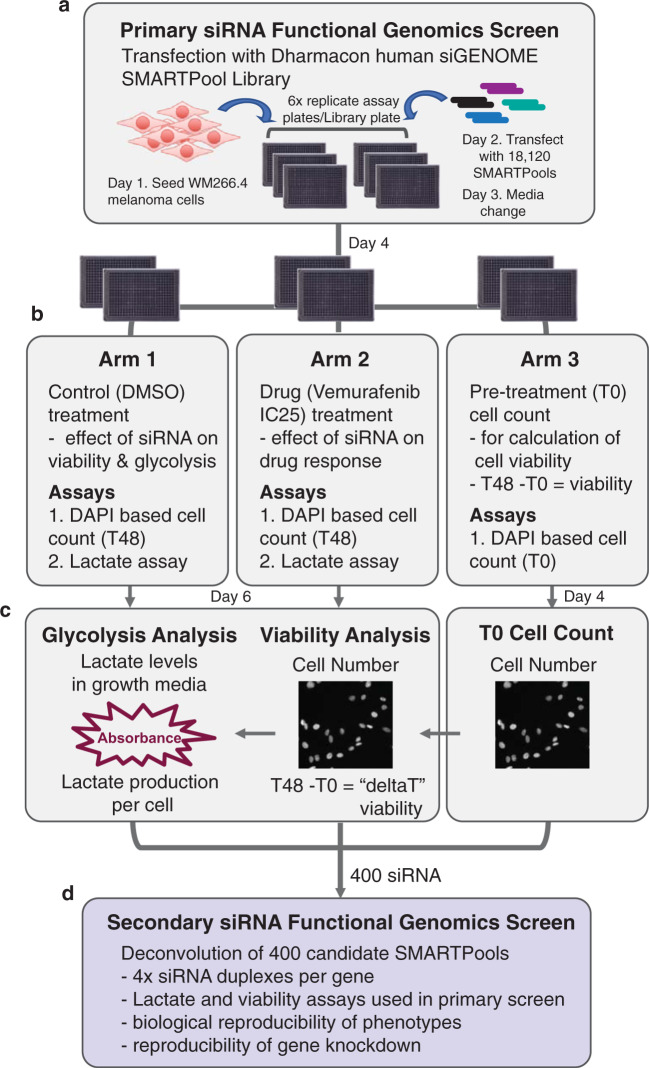
Overview of the multiparametric genome wide siRNA screening approach. (**a**) Experimental scheme for the genome wide siRNA screen to identify genes that regulate cell viability and glycolysis, in the presence and absence of the BRAF inhibitor (BRAFi) vemurafenib. WM266.4 cells were forward transfected with siRNA SMARTpools 72 h prior to assessment for glycolytic capacity and cell viability. The primary screen assessed siRNA SMARTpools targeting 18,120 protein-coding genes (see Data Record 1^11^). (**b**) The screen consisted of 3 arms: 1. Control (DMSO) treatment arm to assess effect of gene knockdown on cell viability and glycolysis, and identify BRAFi specific effects; 2. Drug (vemurafenib) treatment arm to assess effect of gene knockdown on BRAFi response; and 3. Pre-treatment arm to calculate cell number prior to treatment (T0) for calculation of cell viability (T48 – T0 = deltaT). (**c**) Functional assays used to determine lactate production per cell and cell viability, in the presence or absence of BRAFi. Automated image analysis was used for cell nuclei counts to determine cell number. The lactate assay is an enzymatic based assay performed on growth media, and absorbance values were normalised to cell number to calculate lactate production per cell. Wells that were identified as ‘Low Cell Density’ were excluded from lactate analysis. (**d**) 400 candidate siRNA SMARTPools were selected for validation in a secondary deconvolution screen, whereby the 4 individual siRNA duplexes that comprise the SMARTPools were arrayed into individual wells (see Data Record 2^12^).

A multiparameter functional genomics screen using a genome-wide protein-coding RNAi library was designed to identify regulators of the viability and glycolytic response to BRAFi. Lactate production is commonly used to assess glycolysis and can be readily determined in growth media using a lactate dehydrogenase (LDH) enzyme-based assay. Lactate measurements were normalised to cell number, which was determined from nuclear DAPI staining using automated high content image analysis. For the screen, cells were seeded on day 1 and transfected 24 h post-seeding with the Dharmacon human siGENOME SMARTpool library on day 2. On day 3 media was changed, and on day 4 cells were treated with either vemurafenib or vehicle control (DMSO) or fixed and stained with DAPI (T0 cell count). On day 6, media was collected for the lactate assay and cells were fixed and stained with DAPI to determine cell number (T48 cell count). Therefore the screen contained three arms: 1. the control (DMSO) arm used to determine the effects of gene knock down on viability and glycolysis, and establish those genes with selective activity in BRAFi treated cells; 2. the drug (vemurafenib;used at an IC_25_) arm to identify those genes whose depletion enhanced the effects of vem on viability and/or glycolysis; and 3. a pre-treatment arm used to determine cell number prior to treatment (T0 cell count) in order to calculate cell viability (T48 cell count – T0 cell count; positive values indicate change in proliferation and negative values indicate cell death)(Fig. 1).

This approach yielded four hit lists corresponding to genes crucial to cell viability and glycolysis in steady state, and specifically in the context of BRAFi. Expression of these genes was confirmed in the cell line used for the screen using microarray gene expression profiling. In the absence of drug, viability was significantly reduced (robust Z-score < −1.5, corresponding to a FC < 0.08) by depletion of 622 genes and glycolysis was significantly reduced by depletion of 164 genes (Z-score < −1.66, corresponding to a FC < 0.5). To identify genes that regulate viability and glycolytic response to BRAFi, genes were grouped based on fold change data for each parameter in control versus drug treatment conditions (see methods for details), and this analysis identified 63 genes that enhanced the viability response to vem, and 717 genes that enhanced the glycolytic response to vem. Overall, the hit validation rate in the secondary deconvolution screen was high, ranging from 42–60% in the viability arm of the screen, and 30.8–53.25% for the lactate arm of the screen. Notably, these validation rates generally exceed trends observed in other studies using Dharmacon siRNA libraries^3–8^. The datasets generated by this study provide a resource for analysis of genes involved in regulation of cell viability and glycolysis in BRAF^V600^ melanoma cells, in the presence and absence of BRAF inhibition, and are suitable for computational approaches to identify gene regulatory networks that mediate response to BRAF inhibition in melanoma. These genes may serve as potential novel drug targets for development of new therapies to be used in combination with MAPK pathway targeted therapies.

## Methods

### Cell culture

The WM266.4 BRAF mutant melanoma cell line was purchased from the American Tissue Culture Collection (ATCC), and identity confirmed using STR profiling. WM266.4 cells were maintained in RPMI 1640 (GIBCO) containing 10% FBS (same batch used throughout the screen) and 2mM L-alanyl-L-glutamine in a 37 °C humidified, 5% CO2 incubator. The BRAF and NRAS mutation status of the WM266.4 cell line has been reported previously^9^. The same batch of WM266.4 cells was used throughout the primary and secondary screens. For each set of siRNA library plates screened (58x library plates total; 10–16 library plates screened per week), 4x vials of cells were recovered from liquid nitrogen and cultured for 1x passage prior to use in the screen. For drug treatments, phenol-free RPMI 1640 (GIBCO #11835) media was used, as phenol-red interferes with the colorimetric-based readout of the lactate assay.

### High throughput RNA interference screening

The Dharmacon human siGENOME SMARTpool library (RefSeq. 27; Dharmacon RNAi Technologies, Horizon Discovery) was used for the screen. The library was arrayed in 384-well plate format (58x library plates) and screened within the Victorian Centre for Functional Genomics (VCFG, Peter MacCallum Cancer Centre, Australia). The siRNA library was screened in batches (10–16 library plates screened in groups of 4 plates on the robot deck), and each library plate was assayed in duplicate. Positive and negative siRNA controls were located in columns 2 and 23, and media only controls for the lactate assay were located in column 24. The positive control used for the viability assay was an siRNA SMARTPool targeting polo-like kinase 1 (PLK1; Dharmacon, M-003290-01; 8x wells per plate), a critical mitotic kinase required for cell survival; and for the lactate assay an siRNA SMARTPool targeting pyruvate dehydrogenase kinase 1 (PDK1; Dharmacon, M-005019-00; 8x wells per plate), a key regulator of glucose metabolism. The siOTP non-targeting control (siOTP-NT) SMARTPool (Dharmacon, D-001810-10) was used as a negative control for both screening assays (16x wells per plate). Based on substantially reduced viability when optimising reverse-transfection of the WM266.4 melanoma cells we opted for a forward transfection approach, whereby cells were seeded 24 hr prior to siRNA transfection. This also offered the advantage to ensure cell viability was standardised for every screen session.

All liquid handling steps were performed using a robotic BioTek 406 liquid handling platform (z-height of 36), unless otherwise stated. The siRNA transfection was performed using a Caliper Sciclone ALH3000 liquid handling robot (Perkin Elmer, USA). All fixation and staining solutions were filtered (0.45μm filter) prior to use and plates were briefly centrifuged (500 × g for 30 sec) prior to all incubations. High content imaging of DAPI nuclear staining was performed using a Cellomics ArrayScan VTi automated microscope (Cellomics, Thermo Fisher Scientific, USA) with attached plate handler (Twister II, Caliper Life Sciences). Cell number was determined from images using the Cellomics “Cell cycle” bioapplication (Cellomics, Thermo Fisher Scientific, USA). Lactate concentration in media was determined using an L-lactate assay kit (Eton Biosciences) and absorbance (490 nm) was determined using a Cytation 3 Imaging Multi-Mode plate reader (BioTek) with attached plate handler (BioTek BioStack).

Consumable reagents required for the screen:

- Dharmacon siGENOME siRNA SMARTPool library RefSeq. 27. This corresponds to 18,120 SMARTPool reagents arrayed across 58 library plates.
- RNAi MAX transfection lipid (Invitrogen).
- Opti-MEM (Gibco, #31985062).
- 384-well, black-walled, tissue-culture treated plates, with clear flat bottom wells, sterile, with lid (Corning Cat # CLS3764).
- V-bottom 384 well plates (Greiner Cat #781280).
- Plateloc adhesive aluminium seals (Agilent)
- Phenol-free RPMI 1640 (GIBCO #11835)
- DMSO (Invitrogen)
- Vemurafenib (vem) (Plexxikon Inc., Berkeley, CA, USA)
- 16% paraformaldehyde (PFA; Electron Microscopy Sciences, USA).
- PBS (Invitrogen).
- Triton-X-100 (Sigma).
- DAPI DNA dye (Invitrogen).
- L-lactate assay kit (Eton Biosciences #1200014002).
- 0.5 M Acetic acid (Sigma).

### Work flow of the screen

#### Day 1: Cell seeding

Cells were prepared for screening by washing in PBS, followed by incubation in 0.25% Trypsin-EDTA (Gibco, #25200-072) for 5 min at 37 °C until detached. Cells were harvested and centrifuged at 500 × g for 5 min, and trypsin-media was removed. The cell pellet was resuspended in growth media, counted and diluted to 18,000 cells per ml, equating to 450 cells per well in 25 μL media. For the screen, each library plate was screened once, using technical replicates of each plate. Therefore, 6x assay plates were required for each library plate: 2x pre-treatment arm (T0), 2x 48 hr control treated arm (0.1% DMSO), and 2x 48 hr drug treated arm (300 nM vem; IC_25_). Cells were robotically seeded into columns 1–23 of the black walled 384-well tissue culture plates (450 cells/well; “assay plates”) in 25 μL growth media. 25 μL of media alone was added to column 24 for the lactate assay background control. Plates were briefly centrifuged (500 × g for 30 sec) and incubated for 10 min on a level bench at room temperature (RT), then incubated overnight at 37 °C in a LiCONiC STX200 automated microplate humidified incubator (37 °C with 5% CO_2_). All plates were handled in the exact same consecutive order at each step of the screen workflow.

#### Day 2: Transfection

siRNA library stock plates and manually prepared control plates were thawed at room temperature 1 h prior to use. Transfections were performed in groups of 4x siRNA library plates. For each group of library plates, RNAi MAX transfection lipid was prepared in Opti-MEM at a ratio of 1 μL:1,250 μL, equating to a final concentration of 0.03 μL lipid in 37.5 μL per well (Table 1). The RNAi MAX-Opti-MEM mix was incubated for 5 min, before 75 μl was dispensed into each well of a V bottom 384 well plate (“complexing plate”) using the BioTek 406 multi-well dispenser. Next, 10.2 μl of siRNA (1 μM) was transferred from the library plates to the complexing plates using the Caliper Sciclone ALH3000 liquid handling robot, mixed, and incubated for 20 min at room temperature. After 20 min, 12.5 μL of lipid/siRNA complexes was added to 6x assay plates containing 25 μL media, giving a final concentration of 40 nM siRNA in 37.5 μL (Table 1). Assay plates were pulse centrifuged to 500 × g, then returned to the automated microplate adapted incubator (37 °C with 5% CO_2_).

**Table 1:** Primary screen transfection protocol at a glance.

Cells/well	Volume media/well	Volume RNAi-Max/well	siRNA concentration	Final vol/well
450	25 μL	0.03 μL	40 nM	37.5 μL

#### Day 3: Media change

Growth media was changed on all assay plates 24 h post transfection using a BioTek 406 liquid handling robot. Aspiration steps were performed using a z height of 36 (4.57 mm above carrier, leaving ~6 ul), to ensure that the cell monolayer was not disturbed, and 25 μL of pre-warmed RPMI media was dispensed into each well. Assay plates were briefly centrifuged (500 × g for 30 sec), then returned to the automated microplate adapted incubator (37 °C with 5% CO_2_).

#### Day 4: Drug treatment and pre-treatment (T0) cell count

##### Drug treatment

Growth media was removed from 4x replicate assay plates as above and replaced with 25 μl of drug (2x plates) or vehicle (2x plates) containing media. Treatment media was prepared using Phenol-free RPMI, containing 10% FBS and 2 mM glutamine. 1 μl of DMSO or vemurafenib (300 μM, corresponding to an IC_25_ concentration) was added per mL of media, to give a final concentration of 0.1% DMSO and 300 nM vem. Plates were briefly centrifuged (500 × g for 30 sec) and incubated for a further 48 h.

##### Pre-treatment cell count

Growth media was removed from 2x replicate assay plates and replaced with 40 μl of fixative (4% PFA in PBS). Plates were incubated for 20 min at room temperature prior to aspiration of fixative and dispense of 40 μL of staining solution (1 μg/mL DAPI, 0.2% Triton-X-100 in PBS). Plates were incubated for a further 20 min prior to aspiration of staining solution and dispense of 40 μL PBS. Plates were then sealed, ready for imaging.

##### Imaging

Plates were imaged using a Cellomics ArrayScan VTi automated microscope using a 10x objective and 25 fields were captured per well. Cell number was determined from images using the Cellomics “Cell cycle” bioapplication (Cellomics, Thermo Fisher Scientific, USA). Optimal exposure time and object identification thresholds were identified for each individual batch of screening plates based on 10–15 control wells. Nuclei size thresholds were also applied in order to quantify only healthy nuclei, and these size thresholds were assessed for each batch of screening plates using siPLK1 cell death control wells. This constituted the pre-treatment “T0” cell count.

#### Day 6: Media collection, post-treatment cell count and lactate assay

##### Media collection

For every assay plate, one intermediate plate for dilution of media and one destination plate was labelled. Using the BioTek, 20 μL of PBS was dispensed into each intermediate plate. Prior to media collection, assay plates were briefly centrifuged for 3 min at 500 × g to prevent transfer of cellular debris in the media. 10 μL of growth media was then aspirated from each well of the assay plate and transferred to the corresponding intermediate plate and mixed using the Caliper Sciclone ALH3000 liquid handling robot. 15 μL of 1:3 diluted media was then transferred into the destination plate. The diluted media plates were then sealed and stored at −80 °C. Media plates were processed later the same day in batches of 32 plates.

##### Post-treatment cell count

Growth media was removed and plates were fixed and stained in batches of 16 plates as described above.

##### Imaging

Plates were imaged using a Cellomics ArrayScan VTi automated microscope using a 10x objective and 25 fields were captured per well, with cell number determined as described above. This constituted the T48 cell count. To calculate cell viability, the pre-treatment cell count (T0) was subtracted from the post-treatment cell count (T48) to generate the parameter “deltaT”, that reflects the change in cell number throughout the drug treatment.

##### Lactate assay

Lactate levels in growth media was determined using the Eton Biosciences lactate assay kit following the manufacturer’s protocol that we adapted for automated liquid handling. Lactate reagent and plates containing diluted media were defrosted and equilibrated to room temperature (~3 h). Once defrosted, plates were briefly centrifuged for 3 min at 500 × g. In order to determine lactate concentration in media, and monitor performance of the lactate assay across different batches of screening plates, lactate standard curve plates were prepared. Lactate standard curves were prepared in new 384 well plates by adding 0–3.2 mM lactate standards to quadruplicate wells, and a new lactate standard plate was run every 32 screening assay plates. To run the assay, 15 μL of lactate reagent was dispensed into one media plate at a time using the Biotek, at 3 min intervals. This was to ensure an equal reaction time for each plate due to the time-sensitive catalytic nature of the assay. The plate was immediately centrifuged to 500 × g, mixed on an orbital shaker for 20 sec, and then placed in a non-CO_2_ 37 °C incubator for 45 min. The reaction was quenched by adding 15 μL of acetic acid (0.5 M), followed by immediate centrifugation to 500 × g and mixing on an orbital shaker (20 secs).

##### Absorbance detection

Absorbance (490 nm) was determined using a Cytation 3 Imaging Multi-Mode plate reader (BioTek) with attached plate handler (BioTek BioStack). To calculate lactate concentration, absorbance values were fitted to the equation generated from the lactate standard curve. The average value of all media only wells on each plate (column 24) constitutes background lactate levels in growth media and was subtracted from all assay wells. The lactate per cell parameter was determined by normalizing the background subtracted lactate concentration to the corresponding T48 cell count for that well.

### Primary screen data analysis

**Fig. 2:**
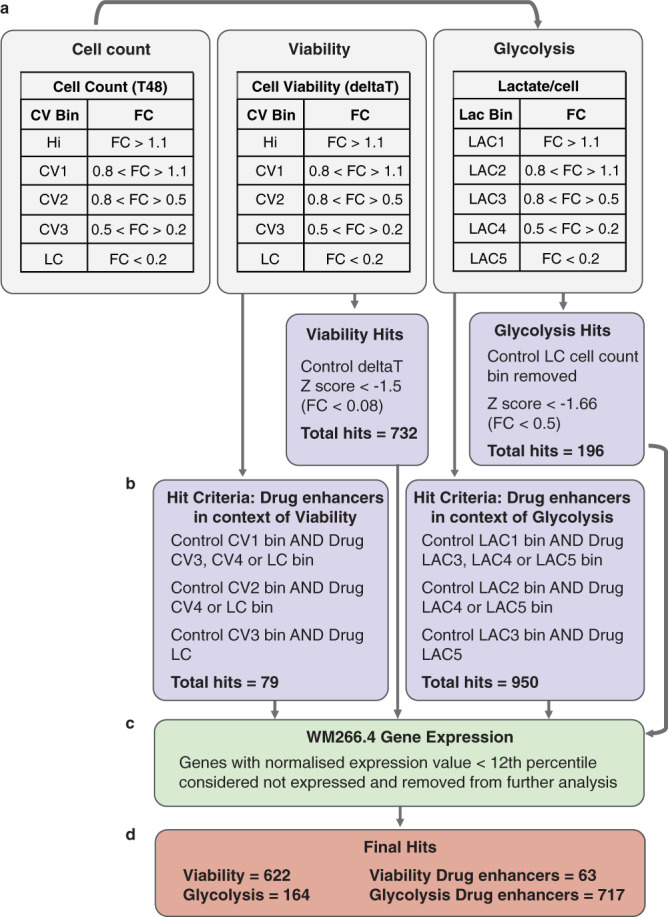
Hit classification strategy applied to the genome wide primary screen. Genes were classified according to a binning strategy for each of the screen output parameters to identify regulators of cell viability and glycolysis (**a)**. Control and drug hit bins were then applied to identify genes that enhance drug effects on viability and glycolysis. Genes that satisfied the listed control and drug bin criteria were classified as drug enhancer hits. (**b)**. Putative hits were triaged for expression in the WM266.4 cell line **(c)**, resulting in 622 viability hits, 164 glycolysis hits, 63 viability drug enhancer hits and 717 glycolysis drug enhancer hits **(d)** (FC = fold change; Hi = high count; CV = cell viability; LC = low count; LAC = lactate).

A binning strategy was developed for each of the screen output parameters to allow identification of SMARTPools that elicited effects in the control or drug only arms of the screen (Fig. 2). This facilitated identification of genes whose depletion perturbed viability or glycolysis, or selectively enhanced the effects of vem on viability and glycolysis, which was the primary aim of this study.

#### Cell number

T48 cell count data were expressed as fold change (FC) relative to the average of all siOTP-NT control wells included on each plate (n = 16), and normalised T48 cell count values were averaged across replicate plates. Robust Z-scores, a statistical measure incorporating the median and the median absolute deviation (MAD) of the samples^10^ were also calculated. Genes were classified into bins based on their effect on cell number in control (steady state) and drug treated conditions (Fig. 2). These bins were primarily used for analysis of the lactate per cell parameter in order to identify regulators of glycolysis (see below).

#### Cell viability

To determine what genes affect cell viability (CV), the change in cell number during treatment (defined as “deltaT”) was calculated from the pre-treatment (T0) and post-treatment (T48) control cell counts (T48 – T0 = deltaT). The deltaT values were normalized to the average of all siOTP-NT control wells included on each plate, and the average deltaT FC value across replicate plates and the robust Z-score were determined. This constituted the viability parameter of the screen. Genes were classified into bins based on their effect on cell viability in control (steady state) and drug treated conditions. Viability hits were determined as genes with a robust Z-score < −1.5 (corresponding to a FC < 0.08) in the control arm of the screen (Fig. 2a). We chose a less stringent cutoff than what is commonly applied to siRNA screens (Z-score cutoff of +/−2 commonly applied to siRNA screens), as we were interested in applying a pathway/network enrichment approach to analysing these data to account for variability in siRNA knock down efficiencies (for example, some genes may individually score low because of gene knockdown efficiency, however function in a pathway enriched in the dataset and therefore be of potential interest). This approach resulted in identification of 732 viability hits.

#### Glycolysis

To determine what genes affect glycolysis, the lactate per cell parameter was normalized to the average of all siOTP-NT control wells included on each plate. The average lactate per cell FC value and robust Z-score were then calculated across replicate plates. Due to inaccuracies in lactate quantitation at low cell number, lactate per cell data was filtered based on T48 cell count to remove genes within the control LC bin (FC < 0.2). Genes were classified into bins based on their effect on glycolysis in control (steady state) and drug treated conditions. Glycolysis hits were determined as genes with a robust Z-score < −1.66 in the control arm of the screen (corresponding to a FC < 0.5; control LAC4 and LAC5 bins)(Fig. 2a). This approach resulted in 196 glycolysis hits.

### Identifying drug enhancers

#### Cell viability

To determine what genes enhance the effect of drug used at an IC_25_ concentration on cell viability, genes were filtered according to both control and drug cell viability (deltaT) hit bins (Fig. 2a). Hits were identified as follows: control (c) Hi AND drug (d) CV2, dCV3 or dLC; cCV1 AND dCV3 or dLC; cCV2 AND dLC (Fig. 2b). This approach resulted in identification of 79 drug enhancers in the context of viability.

#### Glycolysis

To determine what genes enhance the effect of drug used at an IC_25_ concentration on glycolysis, genes were filtered according to both control and drug glycolysis hit bins (Fig. 2a). Genes were first filtered on T48 cell count data to remove genes in the LC cell count bin (FC < 0.2), due to inaccuracies of the lactate assay at low cell number. Hits were identified as follows: control (c) LAC1 AND drug (d) LAC3, dLAC4 or dLAC5; cLAC2 AND dLAC4 or dLAC5; cLAC3 AND dLAC5 (Fig. 2b). This approach resulted in identification of 950 drug enhancers in the context of glycolysis.

### Determination of the WM266.4 steady state gene expression profile

To triage our primary screen hit list we confirmed the expression of candidate genes identified by the screen in the WM266.4 cell line. The WM266.4 steady-state gene expression profile was determined using microarray analysis. Briefly, RNA was extracted from exponentially growing WM266.4 cells using RNeasy columns (Qiagen) as per manufacturer’s directions. RNA was hybridized to a human gene expression array (Agilent) and batch-corrected, normalised gene expression values were determined. The median, 25^th^, and 75^th^ percentile expression values were calculated, and genes with an expression value below the 12^th^ percentile (normalised expression value of 15.1) were considered not expressed and removed from further analysis. This resulted in: 110 genes removed from the viability (deltaT cell count) hit list, 32 genes removed from the glycolysis (lactate per cell) hit list, 16 genes removed from the viability drug enhancer hit list, and 233 genes removed from the glycolysis drug enhancer hit list (Fig. 2c). The final number of genes classified as hits for each parameter of the screen are listed in Fig. 2d.

### SMARTPool deconvolution validation screen

In order to validate hits identified in the primary genome-wide screen, the four individual siRNA duplexes comprising each SMARTPool were arrayed into individual wells. This allows reproducibility of the reagents and the gene knockdown phenotypes to be assessed. Screen methodology and functional assays were the same as that used for the primary screen. The only modification was the transfection protocol, whereby individual siRNA duplexes were screened at a final concentration of 25 nM. Given the primary goal of the screen was to identify genes whose depletion enhanced the effect of the drug on viability and glycolysis, we focused on these genes for the deconvolution validation screen. We selected 400 of these genes for validation based on a range of parameters: 300 top ranked drug enhancers for glycolysis and 50 top ranked drug enhancers for viability, where enhancer hits (as defined above and in Fig. 2b) were ranked based on FC and Z-score values for the viability and lactate/cell parameters in the drug arm of the screen; and 50 additional genes based on pathway enrichment analysis and *a priori* knowledge of genes of potential interest to the underlying biology of the screen.

#### Deconvolution screen analysis

The cell number, viability and lactate per cell parameters of the screen were analysed as described above using identical timepoints, cell numbers and drug concentrations. Validation thresholds were initially set based on the fold change values associated with the binning strategy applied to the primary screen, and genes were considered successfully validated if 2 or more siRNA duplexes satisfied these criteria. This approach proved valid for the cell number (T48 cell count) and cell viability (deltaT) screen parameters, based on consistency of assay performance across both screens. We noted, however, that performance of the lactate assay differed between the two screens, whereby the dynamic range (difference between positive and negative lactate standard controls) of the secondary screen was reduced when compared to the primary screen (average 36% less absorbance for 3.2 mM lactate standards in the deconvolution screen, whereas the no lactate background controls differed by only 2.7%). This was likely due to batch variation in enzymatic components of the assay. Given the reduced dynamic range of the assay would affect fold change values of hits in the deconvolution screen, classification of hit validation based on the binning strategy applied to the primary screen would be inaccurate and produce an unacceptable number of false negative hit validations. We therefore revaluated the criteria used to assess validation of drug enhancer hits in the context of glycolysis, and utilised the average drug lactate/cell value normalised to control as a measure of drug enhancement (drug lactate per cell/control lactate per cell). Hits were assessed based on the mean and standard deviation of siOTP-NT negative controls, which is an approach commonly employed in other siRNA screens^10^. Genes were considered validated if 2 or more siRNA duplexes produced a fold change value less than 2x standard deviations from the mean of siOTP-NT controls, which equated to a drug/control lactate per cell ratio of 0.55. Table 2 displays the frequency of genes validating with different numbers of siRNA duplexes for each assay parameter in the control and drug treated arms of the deconvolution screen.

**Table 2:** siRNA duplex validation results in the secondary deconvolution screen.

Screen Output Parameters	siRNA Duplex Validation					Phenotype Confirmed
	0/4	1/4	2/4	3/4	4/4	
Control cell count (T48) bin	83	149	104	52	12	168
Percentage	21%	37%	26%	13%	3%	42%
Drug cell count (T48) bin	61	133	123	65	18	206
Percentage	15.2%	33.2%	30.8%	16.2%	4.5%	51.5%
Control viability (DeltaT) bin	89	112	81	79	39	199
Percentage	22.2%	28%	20.2%	19.8%	9.8%	49.8%
Drug viability (DeltaT) bin	66	94	85	104	51	240
Percentage	16%	24%	21%	26%	13%	60%
Control lactate/cell bin	137	140	78	30	15	123
Percentage	34.2%	35%	19.5%	7.5%	3.8%	30.8%
Drug lactate/Control lactate per cell ratio < 0.55	183	4	121	71	21	213
Percentage	45%	1%	30.25%	17.75%	5.25%	53.25%

## Data Records

### Data record 1

The primary siRNA screen data set is available at PubChem under the accession number AID: 1508588^11^. We provide screen-wide raw data, normalised data, robust z-scores and the results of binning strategies. Samples are defined by siRNA catalogue number (Dharmacon) and Entrez Gene ID.

### Data record 2

The secondary deconvolution screen data set is available at PubChem under the accession number AID: 1508587^12^. We provide screen-wide normalised data, and the results of binning strategies. Samples are defined by siRNA catalogue number (Dharmacon) and Entrez Gene ID.

## Technical Validation

### Replicate plate reproducibility

**Fig. 3:**
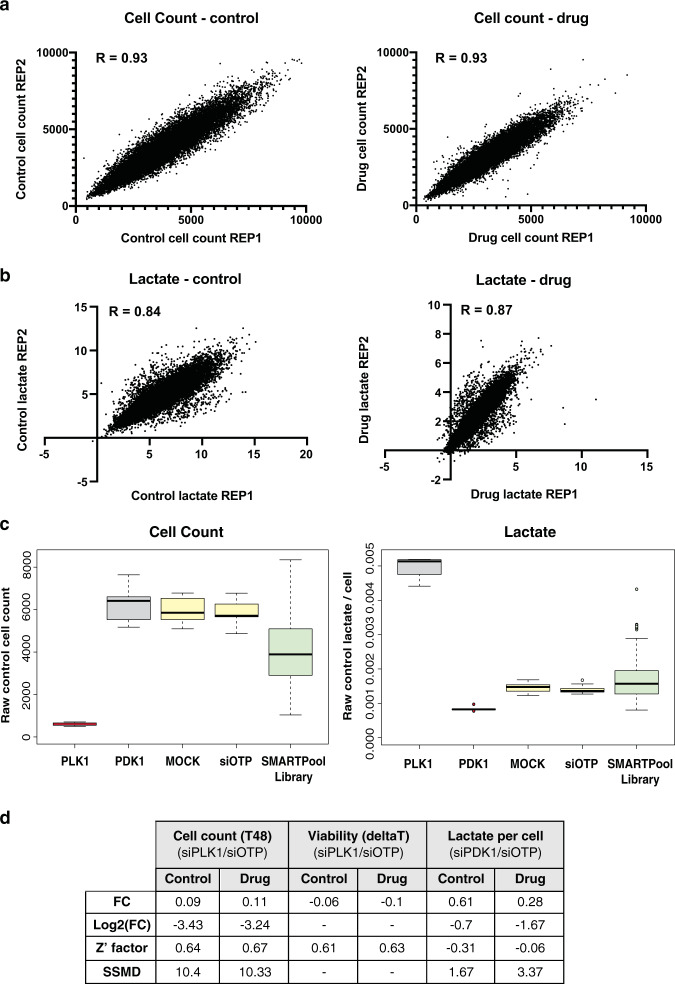
Screen performance. Screen reproducibility was assessed for cell number and lactate values obtained throughout the primary genome wide screen using correlation analysis. Raw cell number **(a)** and lactate values **(b)** were plotted for all siRNA library samples from replicate assay plates, in both control (DMSO) and drug (vem) treatment conditions. The Pearson Correlation Co-efficient is displayed for each comparison. (**c**) Box plots showing distribution of positive (red) and negative (yellow) controls for each assay, and all siRNA library samples (green), generated throughout the screen. Note, knock down of genes that induce extensive cell death, such as viability positive control PLK1, cause spurious high lactate values due to release of cellular contents upon death. These genes were removed from lactate candidates during analysis (see text for details). (**d**) Summary of average fold change (FC), Log2(FC), Z’ factor and strictly standardized mean difference (SSMD) values calculated for the indicated set of assay controls for each assay parameter across the primary screen. See text for details.

The genome-wide siRNA library was screened once for each treatment arm, with technical replicates for each condition; therefore 2x replicate plates for T0 cell count, 2x replicate plates for control treatment (T48 cell count and lactate) and 2x replicates for drug treatment (T48 cell count and lactate). Reproducibility of technical replicate plates was calculated for each set of duplicate plates using correlation analysis. Importantly, replicate plates were highly correlated for both the cell number and lactate production assay parameters throughout the entire screen; the Pearson Correlation Co-efficient for both the control and drug cell count was 0.93 (Fig. 3a), and for lactate was 0.84 in control and 0.87 in the drug arm of the screen (Fig. 3b).

### Control performance

The primary screen was performed over 4 weeks and to ensure high fidelity, the performance of siOTP-NT controls was assessed for each assay parameter on each plate to ensure they were concordant. Any control that scored a coefficient of variance higher than 30% would be considered a failed plate. The distribution of positive and negative controls for each assay throughout the screen are visualised in Fig. 2c. Performance of positive and negative controls was quantified using the Z’ factor (Fig. 3d), a statistical measure of the dynamic range between positive and negative controls that encompasses the mean and standard deviation of each control^10^. Generally, a Z’ factor of >0.1 is considered acceptable for high throughput siRNA screens, however it is worth noting that this statistic assumes each control is at least 3 standard deviations from each other, therefore Z’ factors do not perform well for moderate strength positive controls^10^. Assay controls for cell number in the control and drug treated arms of the screen performed strongly, with an average siPLK1/siOTP-NT Z’ factor of 0.60 and 0.67, respectively (Fig. 3d; calculated as average Z’ for each individual plate, across the whole screen). Average Z’ factors for the cell viability (“deltaT”) parameter of the screen were also very robust, with 0.61 in the control and 0.63 in the drug arm of the screen. Overall, these data demonstrate excellent performance of the cell number and viability assays throughout the screen. The average siPDK1/siOTP-NT Z’ factor for the lactate assay was much lower at −0.31 for the control and −0.06 for the drug treated arms of the screen, however this result was expected based on the moderate strength of the siPDK1 positive control in the lactate assay (Fig. 3c). Based on these limitations with the moderate strength siPDK1 positive control, we also monitored consistency of FC values throughout the screen as a measure of assay performance (average fold change in control conditions 0.61, and in drug conditions 0.28; Fig. 3d).

A more statistically rigorous measure of assay performance that addresses limitations of moderate strength controls is the Strictly Standardized Mean Difference (SSMD)^10,13^. The SSMD was specifically developed for use with RNAi screening which often rely on moderate strength positive controls, and is calculated as the ratio between the difference of the means of positive and negative controls, and the standard deviation of the difference between the control populations^13^. Note, the SSMD was calculated from Log2 fold change data because this statistical measure assumes a symmetrical distribution of data; this precluded analysis of the deltaT viability parameter in siPLK1 samples due to negative values. High average siPLK1/siOTP-NT SSMD values were calculated for the cell number assay in both the control (10.4) and drug (10.33) treatment arms of the screen (Fig. 3d), again indicating excellent quality of this assay throughout the screen in the context of a very strong positive control^14^. Importantly, the average siPDK1/siOTP-NT SSMD value for the lactate assay was 1.67 in the control, and 3.37 in the drug treatment arms of the screen (Fig. 3d), indicating strong performance of the lactate assay in control and drug treatment arms of the screen in the context of a moderate strength positive control^14^.

### Identification of known regulators of cell viability and glycolysis

The primary screen cell viability assay identified many genes known to be essential for cell proliferation and viability (Data Record 1^11^). The “deltaT” viability metric in the control arm of the screen identified 622 genes that reduced the viability of cells. Notably, these genes included many cell cycle regulators, mRNA translation regulators, and components of the ribosome and proteasome, that are known to be required for viability in many different cellular contexts. As expected, multiple components of the MAPK pathway were also identified, consistent with the BRAF mutation status of the cell line used for the screen.

The primary screen lactate assay also identified known regulators of glycolysis and cellular metabolism (Data Record 1^11^). The lactate per cell parameter in the control arm of the screen identified 164 genes that reduced lactate production per cell. The two top ranked hits were phosphogluconate dehydrogenase (PGD; Entrez ID 5226), the second dehydrogenase in the pentose phosphate shunt that is directly coupled to glycolysis, and pyruvate dehydrogenase alpha 1 (PDHA1; Entrez ID 5160), which provides the primary link between glycolysis and the tricarboxylic acid (TCA) cycle in mitochondria. Amongst the top ranked hits were also dihydrolipoamide S-acetyltransferase (DLAT; Entrez ID 1737), another component of the pyruvate dehydrogenase complex, and 6-phosphofructo-2-kinase/fructose-2,6-biphosphatase 3 (PFKFB3; Entrez ID 5209), a direct regulator of glycolytic flux. Furthermore, BRAF (Entrez ID 673) and MAPK1 (ERK2; Entrez ID 5594), that we have previously shown regulate glycolysis in BRAF mutant melanoma cells^1^, were also amongst these genes.

### Number of genes validating with siRNA duplexes in the deconvolution screen

Table 2 summarises the frequency of genes validating with different numbers of siRNA duplexes for each assay parameter in the control and drug treated arms of the deconvolution screen. Analysis of the deconvolution screen data (Data record 2^12^) for cell number (T48) and viability (deltaT) parameters determined that: 168 genes for control cell count (42%), 206 genes for drug cell count (51.5%), 199 genes for control viability (deltaT; 49.8%), and 240 genes for drug viability (deltaT; 60%), successfully reproduced the primary screen phenotype with 2 or more siRNA duplexes. The validation rate was lower for the control lactate per cell parameter of the screen with 123 genes (30.8%) confirming the original screen phenotype, however we note that genes were not selected for the deconvolution screen based on this parameter. Due to reduced dynamic range observed in the lactate assay in the deconvolution screen compared to the primary screen (discussed above), drug enhancers in the context of glycolysis were assessed based on the drug/control lactate per cell parameter. This analysis confirmed 213 genes as drug enhancers in the context of glycolysis (53.25%). Notably, the validation rates for the primary outputs of the screen (drug enhancers in the context of viability and glycolysis at 60% and 53.25%, respectively) exceeds trends observed in other studies using Dharmacon siRNA libraries^3–8^.

## Usage Notes

The siRNA screening data (Data records 1&2^11,12^) are provided for other researchers to apply different normalisation and binning strategies for identification of viability and glycolysis regulators in both steady-state and drug treated conditions. This study focused on identification and validation of drug enhancer hits in the context of glycolysis as a way to identify new candidate drug targets to improve response to BRAF targeted therapies in melanoma patients. However, the screen dataset also contains genes required for glycolytic capacity and cell viability in steady state conditions and thus provides a rich resource for understanding molecular mechanisms that regulate glycolysis and viability. To the best of our knowledge, this is the first publicly available genome-wide screen for regulators of glycolysis in any human cellular system. The data was analysed using in house pipelines focusing on standard metrics including the Z’factor, Z score and SSMD. There is no specifically relevant code.

## References

[1] Parmenter TJ (2014). Response of BRAF-mutant melanoma to BRAF inhibition is mediated by a network of transcriptional regulators of glycolysis. Cancer Discov.

[2] McArthur GA (2012). Marked, homogeneous, and early [18F]fluorodeoxyglucose-positron emission tomography responses to vemurafenib in BRAF-mutant advanced melanoma. J Clin Oncol.

[3] Falkenberg KJ, Gould CM, Johnstone RW, Simpson KJ (2014). Genome-wide functional genomic and transcriptomic analyses for genes regulating sensitivity to vorinostat. Sci Data.

[4] Adamson B, Smogorzewska A, Sigoillot FD, King RW, Elledge SJ (2012). A genome-wide homologous recombination screen identifies the RNA-binding protein RBMX as a component of the DNA-damage response. Nat Cell Biol.

[5] Simpson KJ (2008). Identification of genes that regulate epithelial cell migration using an siRNA screening approach. Nat Cell Biol.

[6] Brass AL (2008). Identification of host proteins required for HIV infection through a functional genomic screen. Science.

[7] Smith JA (2010). Genome-wide siRNA screen identifies SMCX, EP400, and Brd4 as E2-dependent regulators of human papillomavirus oncogene expression. Proc Natl Acad Sci USA.

[8] Williams SP (2017). Systematic high-content genome-wide RNAi screens of endothelial cell migration and morphology. Sci Data.

[9] Ikediobi ON (2006). Mutation analysis of 24 known cancer genes in the NCI-60 cell line set. Mol Cancer Ther.

[10] Birmingham A (2009). Statistical methods for analysis of high-throughput RNA interference screens. Nat Methods.

[11] Smith LK (2020). NCBI PubChem Bioassay.

[12] Smith LK (2020). NCBI PubChem Bioassay.

[13] Zhang XD (2007). A pair of new statistical parameters for quality control in RNA interference high-throughput screening assays. Genomics.

[14] Zhang XD (2008). Novel analytic criteria and effective plate designs for quality control in genome-scale RNAi screens. J Biomol Screen.

